# Investigating environmental transmission to resolve a *Bacillus cereus* group outbreak in a neonatal intensive care unit using core genome multilocus sequence typing

**DOI:** 10.1186/s13756-023-01359-0

**Published:** 2024-01-07

**Authors:** Hauke Tönnies, Axel Heep, Jörg Herrmann, Matthias Lange, Alexander Mellmann, Axel Hamprecht

**Affiliations:** 1 Institute of Hygiene, University Hospital Münster, Münster, Germany; 2 Department of Pediatrics, Elisabeth Children’s Hospital, University of Oldenburg, Oldenburg, Germany; 3 Institute of Hygiene, University Hospital Oldenburg, Oldenburg, Germany; 4 Institute of Medical Microbiology and Virology, University Hospital Oldenburg, Oldenburg, Germany

**Keywords:** *Bacillus cereus*, *Bacillus cereus* group, Outbreak, cgMLST, WGS

## Abstract

**Background:**

We analyzed an outbreak of *Bacillus cereus* group (Bcg) at a single-center neonatal intensive care unit level IV by conducting comprehensive sampling of both patients and the environment.

**Methods:**

Between 06/2020 and 10/2021, all Bcg isolates identified by both regular colonization screening and additional sampling of the environment were subjected to whole-genome sequencing, followed by in vitro extraction of MLST ST, resistance genes and virulence factors. Using publicly available genome sequences, we defined an ad hoc core genome multilocus sequence typing (cgMLST) scheme comprising 2759 target genes for Bcg typing, which we applied to the detected isolates. We have compared the results with a stable cgMLST that was published in the meantime and completed the investigation with a SNP analysis.

**Results:**

We analyzed 28 Bcg isolates from patient and environmental samples using MLST and cgMLST. This revealed multiple sequence types, with ST127 being the most common (n = 13). Both cgMLST schemes grouped ten of the 13 ST127 isolates into a cluster, including two invasive isolates from two different patients and several environmental samples. SNP analysis postulated a screen from a ventilation machine as a possible reservoir.

**Conclusion:**

In sensitive settings such as neonatal intensive care units, considering the environment in outbreak analyses is crucial, especially when investigating potential transmission routes through shared devices. When dealing with widespread bacteria such as Bcg, high-resolution typing techniques are necessary. In this study, we successfully resolved an outbreak of Bcg infections using a custom cgMLST scheme combined with a SNP analysis.

**Supplementary Information:**

The online version contains supplementary material available at 10.1186/s13756-023-01359-0.

## Background

The species of the *Bacillus cereus* group (Bcg), also known as *B. cereus* sensu lato, are gram-positive, spore-forming environmental bacteria closely related in phylogeny, belonging to the phylum Firmicutes.

Using methods commonly employed in clinical microbiology, such as matrix-assisted laser desorption ionization-time of flight mass spectrometry (MALDI-TOF MS), it is impossible to distinguish between members within the Bcg [1]. With the increasing availability of sequencing data, the nomenclature based on genomic data has become increasingly complex. A recent review discussing the taxonomy of Bcg has been published [2], and the same group proposed a new taxonomic nomenclature [3].

An unambiguous nomenclature, especially concerning detections in hospital or outbreak settings, remains crucial since the pathogenic potential of members within Bcg varies significantly. The most well-known members of the group, *B. anthracis* and *B. cereus* s.s., have been identified as causative agents for various infections. *B. anthracis* is best known for causing anthrax, while *B. cereus* s.s. is commonly associated with food-borne infections but is also occasionally responsible for septicemia, endophthalmitis, pneumonia, endocarditis, meningitis, and encephalitis, especially in immunosuppressed individuals such as neonates [4–10]. Due to the difficulty in distinguishing members within the group, it is highly probable that infections attributed to *B. cereus* s.s. or *B. anthracis* were caused by other members of the Bcg.

Some nosocomial Bcg outbreaks and clusters in neonatal units have been described in the literature, for example, due to contaminated ventilator circuits or hospital linens [11–17], emphasizing the need to identify potential sources and transmission routes in the environment if clusters of Bcg infections are recognized in a hospital.

The ubiquitous presence of this pathogen, the complex nomenclature and the highly conserved genomes demand a high-resolution typing method to accurately identify the source of a possible outbreak and routes of transmission within a given setting and to delineate cluster-related from unrelated isolates. Traditional typing methods include pulsed-field gel electrophoresis (PFGE) and fluorescent amplified fragment length polymorphism (AFLP) as the main tools [18], which have also been used to analyze Bcg outbreaks [12, 15], but due to technological advances, it is currently common to use whole-genome sequence (WGS)-derived typing to analyze outbreaks of complex species such as Bcg, which also provides relevant information concerning virulence factors and resistance genes.

In general, extracting typing information from WGS data is usually either based on single nucleotide polymorphisms (SNPs) after mapping of read data on reference genomes or based on hundreds to thousands of predefined target genes, which results in core genome multilocus sequence typing (cgMLST) schemes [19–21]. Analyzing clusters of Bcg using SNPs has been described in the literature [14], but to the best of our knowledge, no nosocomial Bcg outbreak analysis using cgMLST has been published yet. Although a stable cgMLST scheme was recently proposed [22], at the time this analysis was performed, no stable cgMLST scheme was available, which is why we defined and applied an ad hoc cgMLST scheme to analyze a cluster of Bcg infections in a neonatal intensive care unit (NICU). We did, however, repeat the analyze using the published stable cgMLST and compared the results. Additionally, we performed a SNP analysis.

## Methods

### Clinical setting

Between June 2020 and October 2021, a total of 340 patients were admitted to a neonatal intensive care unit (NICU) at a university hospital in Germany, which is a tertiary care hospital with approximately 830 hospital beds. As part of the regular surveillance on a high-level NICU, all neonates undergo weekly screening for anal or nasopharyngeal colonization by various pathogens (e.g., Enterobacterales, *Listeria monocytogenes*, *Streptococcus agalactiae,* etc.). Due to an elevated number of *Bacillus cereus* group (Bcg) detections during regular surveillance, swabs or contact plates were additionally used to sample the skin, wounds, and the NICU environment.

### Processing of the isolates

All samples were plated on Columbia sheep blood agar (BD, Heidelberg, Germany) and MacConkey agar (BD) and incubated for 48 h at 37 °C. Species identification was performed using a Biotyper Maldi-ToF system (Bruker, Bremen, Germany). Susceptibility testing was performed by disk diffusion testing according to EUCAST standards. In total, 28 Bcg isolates were detected and included in the study (refer to Table 1). All 28 Bcg isolates were subjected to whole-genome sequencing.

**Table 1:** List of 28 Bcg strains isolated during clinical diagnostic and surveillance efforts

Patient(P)/Environ-mental (E) sample number	Taxon name (BTyper3 based)	MLST ST	Collection date	Specimen	Toxin profile
P1	*B. mosaicus*	127	7/2020	Blood culture	F
P3	*B. mosaicus*	127	6/2021	Wound swab	F
P4	*B. mosaicus*	127	8/2020	Abdomen swab	F
P9	*B. mosaicus*	127	8/2020	Nasal swab	F
P10	*B. mosaicus*	127	8/2020	Nasal swab	F
P11	*B. mosaicus*	127	3/2021	Skin swab	F
P17	*B. mosaicus*	127	8/2021	Nasal swab	F
P18	*B. mosaicus*	127	8/2021	Anal swab	F
E5	*B. mosaicus*	127	8/2020	Swab room sanitizer	F
E6	*B. mosaicus*	127	8/2020	Swab bottle warmer	F
E7	*B. mosaicus*	127	8/2020	Swab room	F
E8	*B. mosaicus*	127	8/2020	Swab screen ventilator	F
E9	*B. mosaicus*	127	8/2020	Swab room	F
P6	*B. mosaicus* subsp*. cereus*	1359	7/2021	Skin swab	D
P14	*B. mosaicus* subsp*. cereus*	26	10/2021	Nasal swab	F
E1*	*B. mosaicus* subsp*. cereus*	144	7/2020	Swab environment	F
E2*	*B. mosaicus* subsp*. cereus*	164	7/2020	Swab environment	F
E3*	*B. mosaicus* subsp*. cereus*	26	7/2020	Swab environment	F
E4*	*B. mosaicus* subsp.* cereus*	144	7/2020	Swab environment	F
P7	*B. mosaicus* subsp. *cereus biovar Emeticus*	26	3/2021	Anal swab	E
P13	*B. mosaicus* subsp. *cereus* biovar Emeticus	26	9/2021	Umbilical swab	E
P16	*B. mosaicus* subsp. *cereus* biovar Emeticus	26	11/2021	Nasal swab	E
P2	*B. cereus s.s*	1331	9/2020	Abdomen swab	A
P5	*B. cereus s.s*	3228	6/2020	Nasal swab	A
P12	*B. cereus s.s*	4	8/2021	Anal swab	A
P15	*B. cereus s.s*	177	10/2021	Nasal swab	A
P19	*B. cereus s.s*	3230	8/2021	Anal swab	A
P8	*B. cereus s.s.* biovar Thuringiensis	3229	2/2021	Anal swab	A

Isolates marked with an asterisk (*) were isolated from an adjacent department. Taxonomic names were assigned using BTyper3 [24], and the toxin profiles were determined in accordance with [30]: A =|nhe^+^ and hbl^+^ and cytK^+^|; D =|nhe^+^ and cytK^+^|; E =|nhe^+^ and ces^+^|; F =| nhe^+^| (refer also to Table 2)

### Whole-genome sequencing

All isolates that arrived at the laboratory before or on 08/21/2020 were sequenced on the Illumina sequencing platform on a single MiSeq instrument (Illumina, San Diego, CA, USA). WGS library preparation, sequencing and subsequent data analysis were performed as described in [23]. Briefly, resulting fastq files were de novo assembled using SKESA. Isolates that arrived after 08/21/2020 were sequenced using the PacBio Sequel II system (Pacific Biosciences Inc., Menlo Park, CA, USA). WGS library preparation, sequencing and subsequent data analysis were performed as described recently [24] with minor modifications. After DNA extraction using the NEB Monarch Genomic Purification Kit (New England Biolabs, Ipswich, Massachusetts, USA), we constructed the sequence library using the SMRTbell Express Template Prep Kit 2.0 (Pacific Biosciences Inc.) in accordance with the manufacturer’s recommendations. After the 15 h-sequencing run on the Sequel II system, the resulting long reads were assembled by applying the “Microbial Assembly” pipeline within the SMRT Link software version 9 (Pacific Biosciences Inc.) using default parameters except for the genome size, which was adopted to 5.4 Mb to reflect the anticipated genome size of Bcg. More details regarding sequencing results are provided in Additional file 6: Table S6.

### Data analysis

We used the BTyper3 tool for taxonomy using a recently proposed taxonomic nomenclature [3, 25], in silico extraction of the MLST ST in accordance with the published scheme at https://pubmlst.org/organisms/bacillus-cereus [25] and virulence factors. We used the SeqSphere^+^ software version 7.0 (Ridom GmbH, Münster, Germany) to create an ad hoc cgMLST scheme, applying it to the 28 isolates (irrespective of the sequencing platform used to generate WGS data) to generate the allelic profiles and subsequent calculation of the distance matrix and the resulting minimum spanning tree (MST). Additionally, we extracted resistance genes using AMRFinderPlus software version 3.11.2 integrated in the SeqSphere^+^ software [26].

### Creation and applying of the ad hoc Bcg cgMLST scheme

To define the ad hoc cgMLST scheme, we screened the NCBI GenBank for a suitable annotated Bcg isolate as seed genome, which served as a starting point for the extraction of potential cgMLST targets, and appropriate penetration genes to filter out infrequent target genes of the seed genome. Utilizing the cgMLST Target Definer tool (version 1.5 with default parameters) in the SeqSphere+ software, we selected all genes from the seed genome that were present in all penetration genomes with a sequence identity greater than 90% and 100% overlap. This formed the basis of the ad hoc cgMLST scheme. Subsequently, we applied this scheme to the outbreak isolates, identifying and assigning a number to each target gene in the isolates to represent the corresponding allele. The combination of these numbers in each Whole Genome Sequencing (WGS) dataset resulted in an allelic profile, which was then used to generate a minimum spanning tree (MST) through pairwise comparisons among all detected isolates using SeqSphere+ . Missing data were disregarded in the pairwise comparisons.

### Applying the stable Bcg cgMLST scheme

Utilizing the recently established cgMLST scheme [22] implemented through the website https://pubmlst.org/ [27], our data sets were uploaded to generate allelic profiles for all 28 isolates via the website's integrated plugin, 'genome comparator.' These profiles were utilized to compute a distance matrix, enabling the creation of a Minimum Spanning Tree (MST). Subsequently, we compared this MST with the one generated using the ad hoc scheme. The output generated by genome comparator including the generated allelic profiles, the distance matrix and more details are provided in Additional file 5: Table S5.

### SNP analysis

We employed the CSI Phylogeny 1.4 web service from the website of the center for genomic epidemiology (https://www.genomicepidemiology.org/) for SNP analysis [28] and to generate phylogenetic trees from our datasets. We used our seed genome as reference. We visualized the generated Newick files using MEGA11 [29] (refer to Figs. 3 and 4).

## Results

### Isolate characteristics

From June 2020 to October 2021, our analysis included a total of 28 isolates from patient and environmental samples, all subjected to Whole Genome Sequencing (WGS) (refer to Table 1). To ensure robustness and minimize the influence of sequencing errors on our findings, all 28 WGS datasets maintained a minimum coverage of 70x (for Illumina) and 100x (for PacBio). Further specifics regarding the sequencing outcomes can be found in Additional file 6: Table S6.

In total, 19 isolates were collected from neonates, primarily through swabs, at various locations: anal (n = 5 isolates), nasal (n = 7), abdomen (n = 3), and skin (n = 2). They were considered colonizations since the patients showed no sign of infections. However, in two cases, the isolates originated from a wound and from a blood culture, and in both cases, an infection was assumed.

The remaining nine sequenced Bcg isolates were obtained from the environment.

### Taxonomy, MLST, virulence factors and resistance genes

Among the 28 Bcg isolates, BTyper3 [3] differentiated *B. mosaicus* (n = 13), *B. mosaicus* subsp. c*ereus* (n = 6), *B. mosaicus* subsp. *cereus* biovar Emeticus (n = 3), *B. cereus* s.s. (n = 5) and *B. cereus* s.s. biovar Thuringiensis (n = 1).

In all isolates, genes coding for sphingomyelinase, non-hemolytic enterotoxins and parts of the BPS capsular polysaccharide were identified. Some isolates also carried genes coding for Cytotoxin K, Hemolysin B, and cereulides. When ingested, these virulence factors can contribute to gastrointestinal symptoms such as diarrhea or emesis. Based on the virulence factors, all isolates were assigned a toxin profile according to [30]. We also detected genes encoding antimicrobial resistance. Only genes with an intact open reading frame where included. Whereas the resistance gene *BcII* was identified in all isolates, the resistance genes *bla1* and *fosB* were additionally found in 15 isolates. Details on the detection of antimicrobial resistance genes are shown in Table 2. Susceptibility testing revealed 100% susceptibility for vancomycin und meropenem and 100% were susceptible with increased exposure (I) for ciprofloxacin in accordance with the breakpoints from EUCAST. Further substances were not tested.

**Table 2:** List of antimicrobial resistance genes and virulence factors found in the Bcg strains

Resistance Gene/Virulence factors	Product	Isolates
*bla1*	Class A beta-lactamase	P2, P5-P8, P12-P16, P19 and E1-E4
*BcII*	Subclass B1 beta-lactamase ≥ BcII family	P1-P19 and E1-E9
*fosB*	Fosfomycin resistance protein fosB	P2, P5-P8, P12-P16, P19 and E1-E4
*cytK-2*	Cytotoxin K	P2, P5, P6, P8, P12, P15, P19
*sph*	Sphingomyelinase	E1–E9, P1–P19
*bps*EH	Part of BPS capsular polysaccharide (sialic acid synthase)	E1–E9, P1–P19
*nhe*ABC	Non-hemolytic enterotoxin	E1–E9, P1–P19
*hbl*ABCD	Hemolysin BL	P2, P5, P8, P12, P15, P19
*ces*ABCD	Cereulide	P7, P13, P16

### Creation of the ad hoc Bcg cgMLST scheme

Overall, the isolates investigated in this cluster suggest a large genomic diversity with various MLST STs observed. However, there was a notable prevalence of ST127 *B. mosaicus* isolates, including the isolate responsible for the bloodstream infection. Consequently, we conducted a search in the NCBI GenBank and selected an annotated Bcg genome with ST127 (as of June 1, 2021) as the seed genome (*Bacillus cereus* strain M3, accession number NZ_CP016316.1). Using the cgMLST Target Definer tool, we identified 5,219 preliminary target genes from the seed genome as potential candidates for a cgMLST scheme. To filter out infrequently occurring genes, we employed six penetration genomes (Accession numbers CP063651.1, NC_003909.8, NZ_CP072766.1, NZ_CP053656.2, NZ_CM001787.1 and NC_018491.1) (see Additional file 4: Table S4 for details). Only genes present in all the penetration genomes were included in the final cgMLST scheme, while all other genes were excluded.

In total, 2264 genes were removed and stored in an accessory gene set, resulting in a final ad hoc cgMLST scheme consisting of 2759 genes (refer to Additional file 1: Table S1).

### Cluster analysis using the ad hoc scheme

**Fig. 1:**
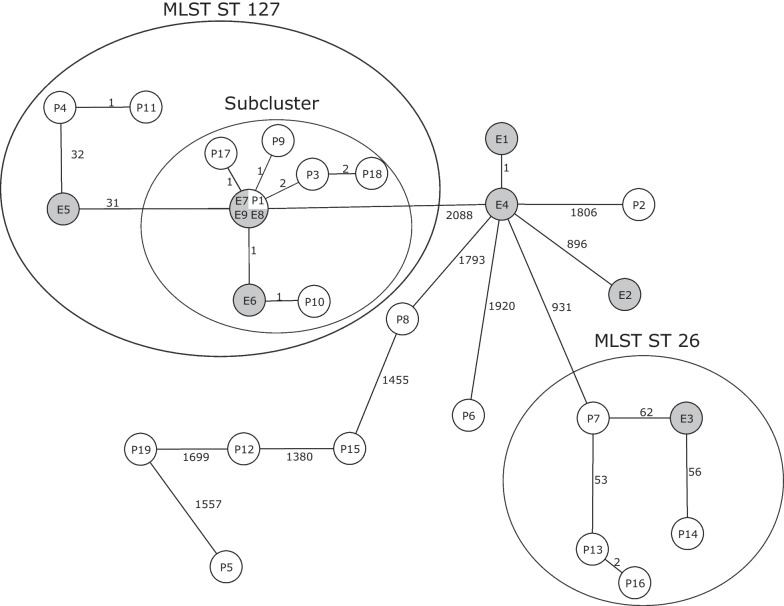
Minimum spanning tree of all 28 isolates based on the allelic profiles of the ad hoc Bcg cgMLST scheme. Each circle represents the genotype based on a unique allelic profile of up to 2,759 cgMLST genes, and the number on connecting lines displays the number of differing alleles in the pairwise comparison. The circles are named with the isolate labels and colored according to their origin (white = Patients, gray = Environment)

To calculate the cgMLST distances between all the isolates and ultimately identify the resulting clusters, we compared the allelic profiles of the 28 isolates (refer to Additional file 2: Table S2) and counted the number of pairwise different alleles. Subsequently, we constructed a *minimum spanning tree* to display the grouping of related isolates (see Fig. 1). Overall, we noted a high genetic diversity comprising allelic differences between 0 and 2100. Within ST127, however, cgMLST grouped ten of the thirteen ST127 isolates into a cluster, where the maximum distance between any of these ten isolates was only five alleles (refer to Additional file 3: Table S3 for the distance matrix), indicating a close relationship. Six of the ten isolates of this cluster originated from clinical samples of different patients (P1, P3, P9, P10, P17, P18), while four isolates had been collected from the immediate patient environment (E6-E9). Of the 10 isolates belonging to this cluster, nine were isolated within a time span of two months at the same ward (Table 1).

Two of the three remaining ST127 isolates (P4 and P11) formed a separate cluster, while isolate E5 was only distantly related to all of them with ≥ 31 differing alleles (Fig. 1).

Within ST26, cgMLST grouped P13 and P16 (two patients who stayed at the same ward) with an allelic distance of 2 together. In contrast, the remaining ST26 isolates showed no close genetic relationship. The closely related environmental isolates E1 and E4 (both ST144) were sampled from an adjacent department. The remaining isolates showed no close relatedness. The clustering based on this cgMLST scheme of all isolates is visualized in Fig. 1.

### Cluster analysis using the stable scheme

**Fig. 2:**
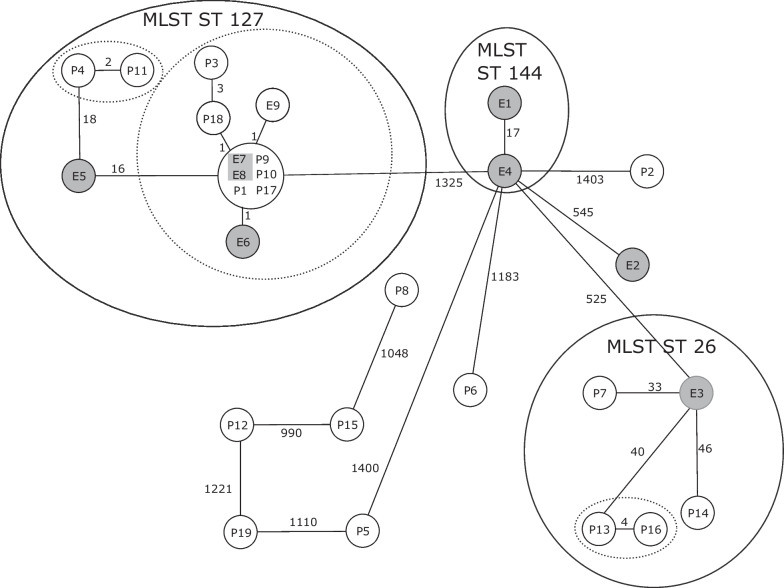
Minimum spanning tree of all 28 isolates based on the allelic profiles of the stable Bcg cgMLST scheme. Each circle represents the genotype based on a unique allelic profile of up to 1568 cgMLST genes, and the number on connecting lines displays the number of differing alleles in the pairwise comparison. The circles are named with the isolate labels and colored according to their origin (white = Patients, gray = Environment)

**Fig. 3:**
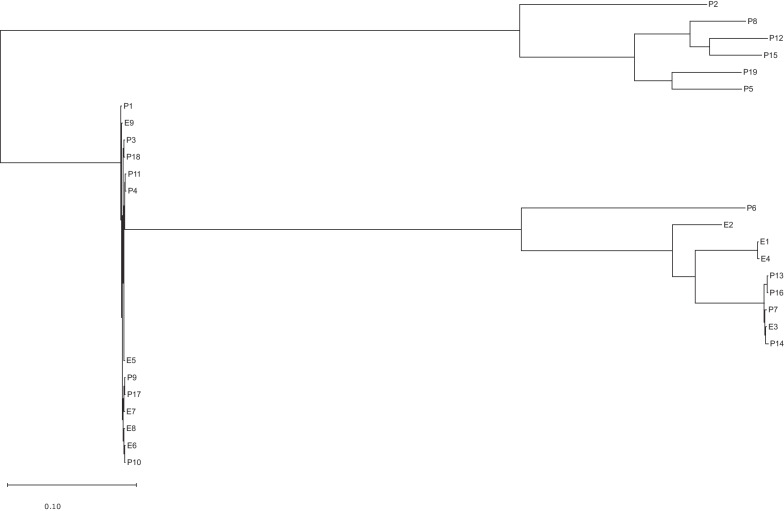
Phylogenetic tree of all 28 isolates based on the SNP analysis. The analysis was conducted using CSI Phylogeny 1.4, accessed through the website of the center for genomic epidemiology (https://www.genomicepidemiology.org/). The resulting tree was downloaded as a newick file and visualized using MEGA11 [29]. The observed topology is in line with the minimum spanning trees from both cgMLST schemes

After the completion of our analysis using the ad hoc scheme, a stable scheme became available (Fig. 2). We reanalyzed and typed the 28 isolates using this stable scheme, comparing allele profiles and constructing a new MST (see Fig. 3) through https://pubmlst.org/ [27].

The stable scheme, employing fewer genes (n = 1568), revealed smaller overall allelic differences (ranging between 0 and 1403) compared to the ad hoc scheme. However, the stable scheme identified similar clusters. Notably, as the only relevant difference, in the stable scheme, isolates E1 and E4 exhibited differences in 17 genes, while the ad hoc scheme detected only a single gene difference between them.

### SNP analysis

To validate the findings from the cgMLST cluster analysis and to gain more insights into potential transmission routes, we utilized the CSI Phylogeny 1.4 web service, accessed through the center for genomic epidemiology's website (https://www.genomicepidemiology.org/), to perform SNP analysis and construct phylogenetic trees based on our datasets [28].

**Fig. 4:**
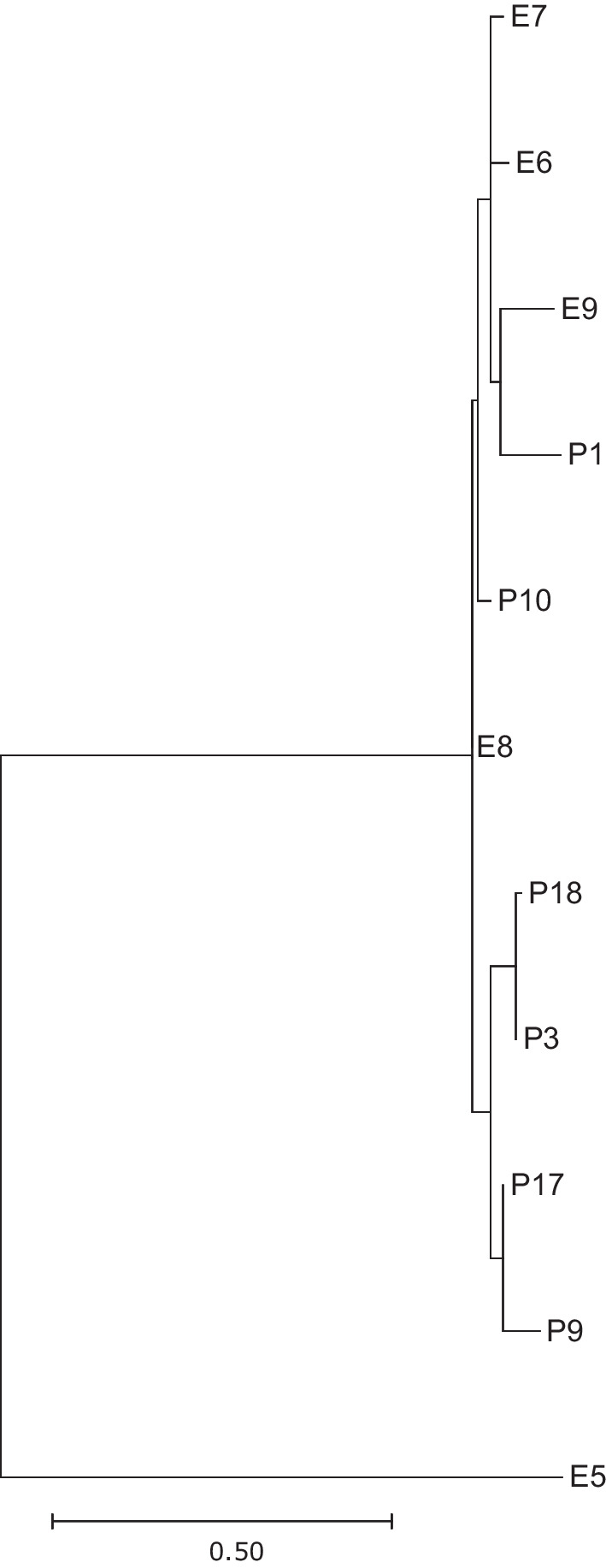
Phylogenetic tree of all but two ST127 isolates based on the SNP analysis. The analysis was conducted using CSI Phylogeny 1.4, accessed through the website of the center for genomic epidemiology (https://www.genomicepidemiology.org/). The resultant tree, focused specifically on ST127 isolates, was downloaded as a newick file and visualized using MEGA11 [29]

The phylogenetic tree, encompassing all isolates (refer to Fig. 3), reflects consistent clustering with the cgMLST results. Specifically focusing on the intriguing ST127 cluster, we conducted a separate SNP analysis excluding outliers P4 and P11. Notably, the tree's topology revealed that the common ancestor of all isolates (except E6) is E8 (refer to Fig. 4).

## Discussion

In the present cluster investigation in the NICU, different Bcg isolates were obtained from various clinical samples, including a blood culture sample, and from the environment. All isolates underwent WGS and were typed using an ad hoc Bcg cgMLST scheme developed specifically for this study. To identify resistance genes and virulence factors, we utilized AMRFinderPlus [26] and BTyper3 [25].

It should be mentioned that detecting a genotypic presence of a resistance or virulence gene does not always correlate with phenotypic expression. Conversely, a specific resistance or virulence might be observed without a genotypic correlate being detected.

While in this case some isolates did possess certain well-known virulence factors, it might be surprising at first glance that the two invasive isolates, which caused the wound infection and the bloodstream infection, contained the fewest virulence factors. This can be explained by the fact that most virulence determinants in *Bacillus cereus* are enterotoxins, which do not play a role in triggering invasive wound or bloodstream infections and usually only cause gastro-intestinal symptoms (e.g. diarrhea, vomiting, nausea) when ingested. As a small side observation, it can be additionally noted that apparently specific virulence factors are not required for Bcg to spread in the environment and to subsequently cause an outbreak.

During the outbreak analysis, it is worth mentioning that a stable and widely accessible cgMLST scheme for Bcg was not available, leading to the utilization of an ad hoc* s*cheme in this study. This ad hoc approach might not have been optimal for the entire Bcg, but it was specifically chosen to address this outbreak scenario. Despite its limitations, this rapid approach effectively resolved the outbreak.

The most critical isolates in the described setting were ST127. The genome NZ_CP016316, as a fully annotated and complete ST127 Bcg genome, seemed quite suitable at the time. We could not anticipate that the genome dataset will be suppressed from RefSeq (apparently due to “unverified source organism”), but still we believe that our analysis was unaffected as the seed genome solely defined targets for similarity searches in other genomes. The sequence of NZ_CP016316 itself is not included in our analysis. The same applies to the suppressed penetration genomes NZ_CM001787.1 and NC_018491.1.

After we had designed the ad hoc scheme and analyzed the isolates, a stable cgMLST scheme was published, giving us the opportunity to compare our ad hoc scheme with the stable scheme. The resulting minimum spanning trees revealed similar topology. However, there is a relevant dissimilarity concerning the allele difference between isolates E4 and E1, which also emphasises the disadvantage of the ad hoc scheme. The allele difference between the two isolates under the ad hoc scheme is only one, which indicates a close genetic relationship. However, 17 different alleles were differentiated under the stable scheme. The subsequent SNP analysis also rather supports the assumption that the two isolates are not as closely related as an allelic difference of one would suggest. Apparently, the selected genes of the seed genome were not representative enough to differentiate these two isolates sufficiently.

However, as the two isolates are outside the relevant ST 127 cluster, this difference has no impact on the interpretation of the outbreak.

Regarding the cluster analysis based on the typing using the two cgMLST schemes and based on the SNP analysis, many isolates exhibited no genetic relatedness, highlighting the ubiquitous nature of this organism. Nonetheless, other isolates displayed close relatedness, indicating potential transmission events between different patients and between patients and the environment.

Among the isolated Bcg strains, a noteworthy set comprised all isolates with ST127, including both invasive isolates from patients P1 and P3. Genetic relatedness analysis using the developed (and stable) cgMLST scheme unveiled a subcluster within this set, consisting of ten out of the 13 isolates (six originating from patients, four from the environment), with a maximum allelic distance of four (see maximum number within the distance matrix comprising of those ten isolates in Additional file 1: Table S3). The remaining three isolates belonging to the same ST exhibited a minimum allelic distance of 31 from any of the isolates within this subcluster. Determining the allelic distance threshold between two isolates in cgMLST schemes poses a complex challenge in outbreak settings [31]. Universally applicable thresholds do not exist, as the allelic difference between two sequenced isolates of the same origin depends on factors such as the time elapsed since transfer, the species' mutation rate, environmental selection pressures, specific cgMLST schemes, and the number of included genes. In the context of this ad hoc developed cgMLST scheme, we consider an allelic distance of at least 31 (respectively 16 in context of the stable scheme) to be sufficiently high to differentiate these three isolates from the described subcluster based on experience and insights gained from studying other species.

Another subcluster that could be more finely resolved by cgMLST was the cluster comprising all ST26 isolates, as only P13 and P16 exhibited an allelic distance of two alleles (respectively four, when applying the stable scheme).

Remarkably, the topology of the phylogenetic tree derived from the SNP analysis suggests that the shared origin of the ten ST127 isolates might be the ventilator screen (sample id E8). Given its tactile operation, it's plausible that this screen facilitated the spread. However, molecular-based typing alone cannot definitively prove this. Nonetheless, this discovery underscores the critical necessity of thorough reprocessing of hand contact surfaces, particularly in sensitive hospital areas.

## Conclusion

In the context of outbreak investigations, especially those involving environmental pathogens within sensitive areas such as a neonatal intensive care unit, it is highly advisable to broaden the scope of analysis to encompass the surrounding environment, since transmission through environmental factors or shared medical devices remains a viable route of infection, although such transmissions can be challenging to detect.

For omnipresent bacteria like Bcg, high-resolution typing techniques such as a stable cgMLST scheme with subsequent SNP analysis prove valuable. However, in the absence of a stable scheme, an ad hoc approach, while having limitations, can still offer insights, as demonstrated in this case.

### Supplementary Information

**Additional file 1: Table S1.** List of core genome genes used for the Bcg ad hoc cgMLST scheme.

**Additional file 2: Table S2.** Allelic profiles of the 28 isolates based on the Bcg ad hoc cgMLST scheme.

**Additional file 3: Table S3.** Distance matrix of the 28 isolates based on the Bcg ad hoc cgMLST scheme.

**Additional file 4: Table S4.** List of Bcg strains used for the ad hoc cgMLST scheme definition.

**Additional file 5: Table S5.** List of core genome genes, allelic profiles and distance matrix based on the Bcg stable cgMLST scheme of the 28 isolates. 

**Additional file 6: Table S6.** Details regarding sequencing results of the 28 isolates.

## Data Availability

All sequence data generated were submitted to the NCBI GenBank under BioProject accession number PRJNA1010155.

## Abbreviations

AFLP: Amplified fragment length polymorphism
Bcg: *Bacillus cereus* group
cgMLST: Core genome multi locus sequence typing
MALDI: Matrix-assisted laser desorption ionization-time of flight mass spectrometry
MLST: Multilocus sequence typing
NICU: Neonatal intensive care unit
PFGE: Pulsed-field gel electrophoresis
SNP: Single nucleotide polymorphism
ST: Sequence type
